# Papillary tumor of the pineal region: analysis of DNA methylation profiles and clinical outcomes in 76 cases

**DOI:** 10.1186/s40478-024-01781-4

**Published:** 2024-07-16

**Authors:** Zhichao Wu, Karen Dazelle, Zied Abdullaev, Hye-Jung Chung, Sonika Dahiya, Matthew Wood, Han Lee, Calixto-Hope G. Lucas, Qinwen Mao, Lorraina Robinson, Igor Fernandes, Matthew McCord, Peter Pytel, Kyle S. Conway, Rebecca Yoda, Jennifer M. Eschbacher, Ossama M. Maher, Martin Hasselblatt, Bret C. Mobley, Jack M. Raisanen, Kimmo J. Hatanpaa, Joshua Byers, Norman L. Lehman, Patrick J. Cimino, Drew Pratt, Martha Quezado, Kenneth Aldape

**Affiliations:** 1 Laboratory of Pathology, Center for Cancer Research, National Cancer Institute, National Institutes of Health, 10 Center Dr., Room 2S235, Bethesda, MD 20892 USA; 2 Division of Neuropathology, Washington University, St. Louis, MO USA; 3 Department of Pathology and Laboratory Medicine, Oregon Health & Science University, Portland, OR USA; 4 Neuropathology Division, Department of Pathology, University of California Davis, Sacramento, CA USA; 5 Department of Pathology, Johns Hopkins University School of Medicine, Baltimore, MD USA; 6 Department of Pathology, Primary Children’s Hospital, Salt Lake City, UT USA; 7 Laboratorio Bacchi, São Paulo, Brazil; 8 Department of Pathology, Northwestern Memorial Hospital, Chicago, IL USA; 9 Department of Pathology, University of Chicago, Chicago, IL USA; 10 Department of Pathology, University of Michigan Medical Center, Ann Arbor, MI USA; 11 Department of Pathology, University of Washington, Seattle, WA USA; 12 Department of Neuropathology, Barrow Neurological Institute, St. Joseph’s Hospital and Medical Center, Phoenix, AZ USA; 13 Division of Pediatric Neuro -Oncology, Pediatric Hematology and Oncology, Kidz Medical Services, Nicklaus Children’s Hospital, Miami, FL USA; 14 Institute of Neuropathology, University Hospital Münster, Münster, Germany; 15 Department of Pathology, Microbiology and Immunology, Vanderbilt University, Nashville, TN USA; 16 Division of Neuropathology, University of Texas Southwestern Medical Center, Dallas, TX USA; 17 Department of Laboratory Medicine, University of California-San Francisco, San Francisco, CA USA; 18 Department of Pathology, Baylor College of Medicine, Baylor Scott & White Medical Center, Temple, TX USA; 19 Surgical Neurology Branch, National Institute of Neurological Disorders and Stroke, National Institutes of Health, Bethesda, MD USA

## Abstract

Papillary tumor of the pineal region (PTPR) is an uncommon tumor of the pineal region with distinctive histopathologic and molecular characteristics. Experience is limited with respect to its molecular heterogeneity and clinical characteristics. Here, we describe 39 new cases and combine these with 37 previously published cases for a cohort of 76 PTPR’s, all confirmed by methylation profiling. As previously reported, two main methylation groups were identified (PTPR-A and PTPR-B). In our analysis we extended the subtyping into three subtypes: PTPR-A, PTPR-B1 and PTPR-B2 supported by DNA methylation profile and genomic copy number variations. Frequent loss of chromosome 3 or 14 was found in PTPR-B1 tumors but not in PTPR-B2. Examination of clinical outcome showed that nearly half (14/30, 47%) of examined patients experienced tumor progression with significant difference among the subtypes (*p* value = 0.046). Our analysis extends the understanding of this uncommon but distinct neuroepithelial tumor by describing its molecular heterogeneity and clinical outcomes, including its tendency towards tumor recurrence.

**Supplementary Information:**

The online version contains supplementary material available at 10.1186/s40478-024-01781-4.

## Introduction

Papillary Tumor of the Pineal Region (PTPR) is a rare neuroepithelial tumor with distinct morphological and molecular features, first reported in 2003 and introduced in the 2007 World Health Organization classification of central nervous system (CNS) tumors [11, 13]. PTPR is generally found within the posterior 3rd ventricle of the brain in both children and adults with a mean age of 33 years [15]. Their common histomorphology includes epithelial-like and papillary growth pattern with expression of S100 and cytokeratins [1, 5, 6, 8, 9, 11]. Glial fibrillary protein (GFAP) immunoreactivity is variable [4]. PTPRs are clinically important to recognize, and included in the differential diagnosis of PTPR are pineal parenchymal tumors, choroid plexus tumors, ependymomas and metastatic adenocarcinomas.

While pathognomonic genomic alterations have not been reported in PTPR, DNA methylation analysis has been shown to be useful in the diagnosis and classification of PTPR as they show a distinct methylation profile compared with other brain tumors [9]. Two PTPR subtypes PTPR-A and PTPR-B have been identified with significant differences in DNA methylation and DNA copy number variations [9]. Chromosome 10 loss has been shown to be characteristic, and other frequent chromosomal alterations include chromosome 3 and 22q losses, chromosome 8 and 12 gains in this tumor class. Moreover, PTPR-A tumors tended to have longer progression-free survival than PTPR-B [9]. To extend the understanding of PTPR, here we report a clinicogenomic analysis based on a cohort of 76 PTPR tumors, all confirmed by DNA methylation profiling, from our clinical experience as well as previously published records.

## Materials and methods

### Sample preparation, DNA methylation profiling and diagnostics

The use of human subject material was performed in accordance with the World Medical Association Declaration of Helsinki and with the approval of the participating Institutional Review Boards. Patient material and clinical data were prepared, and patients were diagnosed as previously described [14]. This study included previously described cases of Papillary Tumor of the Pineal Region as well as cases from the Laboratory of Pathology clinical consult service at the National Cancer Institute (NCI) in Bethesda, MD, USA as well as available methylation array data in the form of raw IDAT files. Samples underwent DNA methylation profiling using Illumina Infinium MethylationEPIC or MethylationEPIC v2.0 array. Publicly available PTPR tumors with methylation profiling were downloaded from GEO database (Additional file 2: Table S1). Tissue histopathology was examined by experienced pathologists involved in clinical diagnosis of these cases.

### Data analysis

DNA methylation profiles were processed using ‘preprocessIllumina’ function from R (version 4.2) minfi package, beta values of samples from different methylation platforms were them integrated by probe names. UMAP was analyzed using non-trivial principal components (n = 13 for all tumors, n = 7 for PTPR only tumors) determined by 1,000 times’ permutation tests. Differentially methylated probes were identified using the Wilcoxon test in R and defined with *p* value < 0.05 and mean beta value difference > 0.2. Hyper-/hypo-methylated gene promoters were defined by more than two significantly hyper-/hypo-methylated probes and zero hypo-/hyper-methylated probes.

Copy number variations (CNV’s) were detected by the conumee package using the same control data set from the CNS tumor classifier [2]. Chromosomal level amplification or deletion was determined manually by the segmentation results using cutoff above 0.1 or below − 0.1. Survival analysis was performed using R survival package after excluding three outlier long time progression-free survival samples (> 150 months). All analyses were performed using R version 4.2.

## Results

### DNA methylation identifies three subtypes of PTPR tumors

To better characterize the PTPR tumors, we combine unpublished PTPR tumors (n = 39) from our clinical consultation DNA methylation practice with published PTPR tumors (n = 37) as a study cohort. Patient demographics for these 76 cases are shown in Additional file 2: Table S1. The median age was 36, of which 28 were pediatric (under age 21) and the remaining 48 cases were adults. There were 40 females and 36 males. Stated tumor location was predominately in the pineal region, although additional descriptions of tumor site were provided, which included surrounding structures, such as third ventricle. Notably, several tumors were described as arising outside of the immediate vicinity of the pineal region, including the cerebellum, brainstem, foramen magnum and thalamus. Most cases received an initial (pre-methylation profiling) diagnosis of PTPR, but some were thought to be ependymoma. Others received a descriptive diagnosis (for example “neuroepithelial neoplasm” or similar) and one case (from the posterior fossa) was given an initial diagnosis of medulloblastoma. Of the 31 cases for which outcomes could be ascertained, 14 cases showed tumor progression and the median progression-free survival (PFS) was 63 months. Eight of these 31 patients died, with a median overall survival of 133 months.

**Fig. 1:**
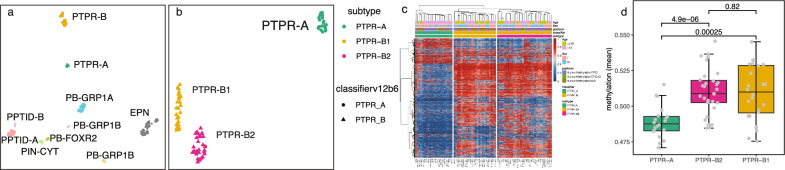
DNA methylation identified three distinct subtypes of PTPR tumors. **a** Unsupervised UMAP plot of collected PTPR tumors and in-house RELA fusion positive supratentorial ependymoma (EPN), and pineal parenchymal and retinal tumors (including PPTID-A/B, PB-GRP1A/GRP1B/GRP2, PIN-CYT). **b**–**c** UMAP plot (**b**) and cluster-heatmap (**c**) of PTPR tumors in this cohort. **d** boxplot of genomic mean methylation across the three PTPR tumor subtypes

To characterize the methylation profiles, we first utilized UMAP as a dimension reduction visualization method to study the methylation patterns of these 76 PTPR tumors with tumors in proximity, including ZFTA fusion-positive supratentorial ependymoma, and pineal parenchymal and retinal tumors (Fig. 1a, Additional file 2: Table S1). UMAP analysis revealed PTPR harboring distinct methylation profiles from other brain tumors and confirmed a clear separation of two known subtypes: PTPR-A and PTPR-B. Notably, we identified a separation of PTPR-B tumors into two groups on the UMAP which suggested subtypes to be refined (Fig. 1a).

To further study and accurately describe the subtypes of PTPR, we analyzed PTPR methylation data without other brain tumors. Principal component analysis varied most (38.4%) in PC1 and clearly separated PTPR-A and -B tumors. PC2 explained only 9.0% of the total variation but was able to distinguish two groups of tumors in PTPR-B. UMAP (Fig. 1b) and cluster-heatmap (Fig. 1c) resulted consistent groups of PTPR tumors and thus we name the tumors into three subgroups/subtypes: PTPR-A, PTPR-B1 and PTPR-B2. We noticed one sample (AB29) that was classified as PTPR-A with high scores but grouped in the UMAP PTPR-B2 tumors, cluster-heatmap showed this sample had features of both PTPR-A and PTPR-B and thus was excluded from follow-up analysis. Mean genomic methylation was significantly (*p* value < 0.001) higher in PTPR-B1 and -B2 compared to PTPR-A, consistent with previous findings [9]. Tumor purity comparison of PTPR-B2 and PTPR-B1 showed no significance (*p* value > 0.60) based on the purity estimation from RF_Purify [10].

We then identified differentially methylated probes across PTPR-A and PTPR-B1/B2 on 41 samples which were profiled using EPIC array. As expected, more probes were hypo-methylated (n = 3,180) in PTPR-A than hyper-methylated (n = 857) when compared to PTPR-B1/B2. We also examined methylation at the gene promoter level and found 196 gene promoters to be hypo-methylated in PTPR-A compared with PTPR-B1/B2, whereas 33 promoters were hypermethylated in PTPR-A compared with PTPR-B1/B2 (Additional file 2: Table S2). We did not appreciate biologically meaningfully associated pathways enriched in the differentially methylated probes. We then examined methylation differences between the PTPR-B1 vs. B2 subtypes and found only small numbers of specific probes that were significantly different between these 2 groups (33 hypermethylated and 17 hypomethylated probes in the B1 versus B2 subtype). Among these changes were 6 promoters that were hypermethylated in the PTPR B1 subtype compared to the B2 subtype, too few for an enrichment analysis.

### Histopathologic characteristics of PTPR

**Fig. 2:**
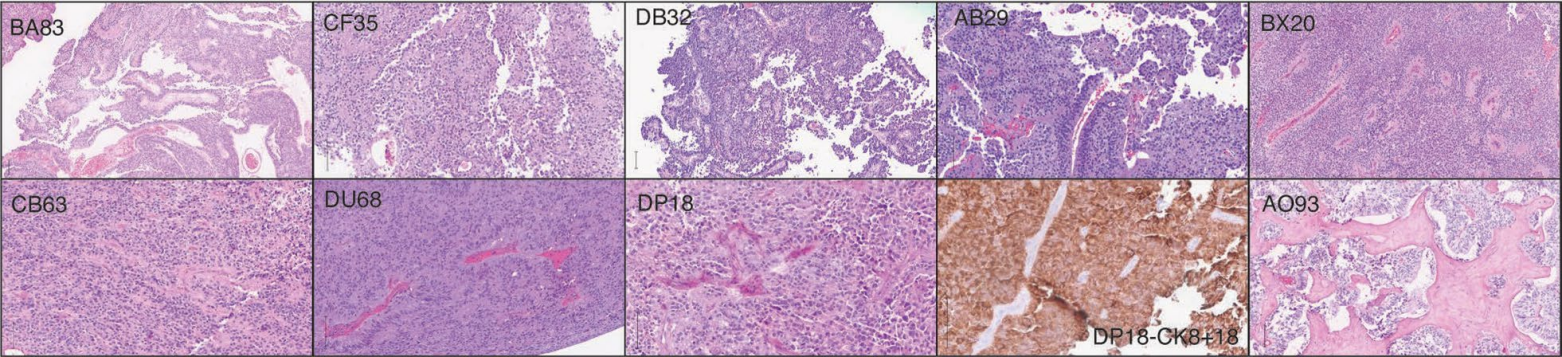
Histopathology of PTPR tumors. Representative histopathologic images of PTPR tumors show a variety of histologic patterns, including prominent papillary architecture (BA83, CF35, DB32) and/or solid morphology with occasional ependymoma-like (AB29, BX20, CB63, DU68, DP18) areas. One case (AO93) showed tumor in association with prominent sclerosis. Tumors were typically positive for cytokeratins, including CK 8/18 (DP 18). Vertical bar in lower left of images = 100 microns

Similar to prior descriptions of this PTPR [8], tumors in our cohort showed an epithelial-like papillary or pseudopapillary growth pattern in which the vessels were covered by layers of columnar or cuboidal tumor cells. Some tumors showed a prominent papillary architecture, while others had a more solid morphology, often exhibiting mixed features (Fig. 2). Most cases received immunohistochemical workup prior to being sent to the NIH for DNA methylation testing and typically showed expression of cytokeratins, including CK18.

### PTPR tumor subtypes exhibited distinct copy number alterations and clinical outcome

**Fig. 3:**
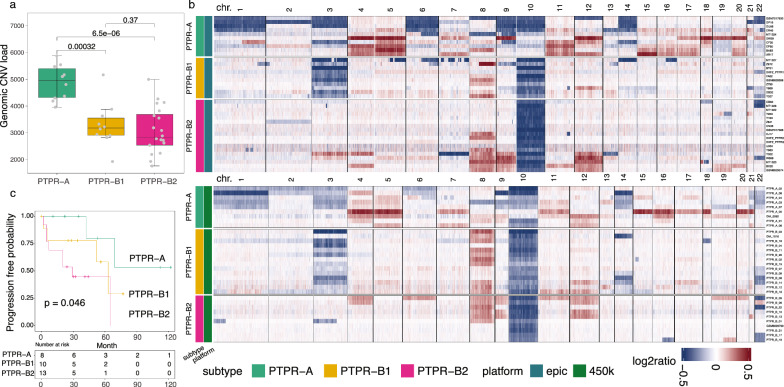
DNA copy number analysis and clinical outcome of PTPR tumors. **a** boxplot of CNV load across PTPR subtypes. **b** CNV heatmap of PTPR tumor samples. **c** progression-free survival of PTPR subtypes

To understand the genetic characters of PTPR subtypes, we then identified copy number variation (CNV) across these tumors. Tumor genomes were often altered by genetic mutation and CNV and might be associated with their biological subtypes. The genomic CNV load was significantly higher in PTPR-A than PTPR-B1 and -B2 (*p* value < 0.0001, Fig. 3a). We determined chromosome level gain or loss based on their CNV profile (Fig. 3b). Chromosome 3/10 loss and chromosome 8 gain was detected in 45.3%, 93.3% and 49.3% of these tumors. In addition, multiple frequent (≥ 30%) chromosomal level CNVs were observed in PTPR-A, including chromosome gains of chromosomes 4, 5, 7, 11, 12, 15, 16, 17, 18 and 20, and losses of chromosomes 1, 3, 6, 9, 10, 14, 19 and 22. Within PTPR-B group, frequent chromosome gains of 8 and loss of 10 were existed in both PTPR-B1/B2. However, chromosome loss of 3 or 14 was frequent in PTPR-B1 tumors (n = 22/24, 7/24) but was not detected in PTPR-2 (n = 0/31, *p* values = 2.21e−13, 6.04e−4, respectively). Of interest, PTPR-B2 showed significantly more frequent gains of chromosomes 9 and 12 (*p* values = 1.52e-3, 4.95e-2, respectively) compared to PTPR-B1.

Since PTPR-B1 and -B2 showed significant differences in their CNV profiles, we examined the methylation clustering/UMAP by removing the effect of chromosomal CNV. We excluded probes from chromosomes 3, 9, 12, and 14 and analyzed the UMAP. In this resulting UMAP plot, PTPR-A, -B1, and -B2 subtypes are still clearly separated (Additional file 1: Fig. S1), indicating that the methylation clustering result was not driven by these CNV differences between the subtypes.

We then investigated the clinical outcome across the three subtypes in cases with available outcome data (Table 1, Additional file 2: Table S1). While the number of cases with available outcomes data was small (n = 30), we note that tumor progression occurred in nearly half of the cases (46.7%, n = 14/30). With this modest sample size, there was some evidence of a relationship between the 3 methylation groups (A vs. B1 vs. B2) and progression-free survival (*p* value = 0.046) (Fig. 3c). Among these tumors, the median PFS times were > 69 months (95% lower confidence limit), 63 months and 29 months for the PTPR-A, -B1 and -B2 subtypes, respectively.

**Table 1:** Summary of PTPR subtypes

	PTPR-A	PTPR-B1	PTPR-B2
# Samples	20	24	31
Patient characteristics			
Age (years, median range)	10–63 (40)	8–52 (26)	1–62 (30)
Sex (male: female)	8:12	11:13	16:15
Histopathology	Epithelial-like papillary or pseudopapillary growth pattern in which the vessels were covered by layers of columnar or cuboidal tumor cells		
Outcome			
Progression (yes/no)	2/6	4/5	8/5
Death (yes/no)	2/6	3/6	3/10
Median survival month [mean (95% confidence interval)]	178	95	77
Months to progression [mean (95% confidence interval)]	NA (0.95UCL = 69)	63	29
Chromosomal alterations (frequency ≥ 30%)			
Gain	4,5,7,11,12,15,16,17,18,20	8	8,9,12
Loss	1,3,6,9,10,14, 19,22	3,10,14	10
Epigenetic alterations			
Hypermethylated genes (promoter)	857	3180	
Mean methylation	0.489	0.51	

Patient characteristics, histopathology features, outcome, DNA copy number variations, and methylation properties across papillary tumor of the pineal region (PTPR) subtypes (n = 75)

## Discussion

PTPR is a rare neuroepithelial brain tumor, with about 200 cases reported to date. Of these, 37 cases have been previously described based on DNA methylation analysis. PTPR patient prognosis has not been well studied, however, it is known that tumor recurrence frequently occurs, necessitating more research on tumor subtype characterization and treatment development. Previous published reports on PTPR have included only morphological descriptions and transcriptional analyses [7, 12]. Heim et al. [9] showed that genomic DNA methylation profiling can effectively distinguish PTPR from other major brain tumor types. PTPR is an important tumor type to be considered in the differential diagnosis of primary CNS tumors. We note that these tumors are most often located in the pineal region, although the stated site of a number of tumors was not necessarily in the pineal region specifically. We note that most of our cases were suspected as PTPR’s prior to methylation profiling, but also note that a subset was given either a descriptive diagnosis or were suspected as an alternative tumor type (for example ependymoma, astroblastoma and medulloblastoma), highlighting the need to increase awareness of this tumor type and also the utility of methylation profiling to evaluate this uncommon tumor type. The consultative nature of the practice that led to this report precluded detailed review of imaging features, however, we note several PTPR cases in our cohort that were reported as cerebellar, or brainstem in origin (Additional file 2: Table S1). In this context, we note a previous report of papillary tumor of the pineal region in the 4th ventricle [3]. We note generally high methylation confidence scores for such cases outside the pineal region (Additional file 2: Table S1), as well as the presence of frequent orthogonal markers (keratin positivity, chromosome 10 loss (not shown)) in these specific cases, further highlighting the importance of this diagnostic consideration, even in the setting of a tumor site described as outside the limits of the pineal region.

Two PTPR subtypes, PTPR-A and PTPR-B, were discovered based on DNA methylation signatures and distinct patterns of CNV. In our study, we extend these findings by further elucidating methylation subtypes and describing correlations with patient outcome. In this study, we find three PTPR DNA methylation subtypes: PTPR-A, PTPR-B1 and PTPR-B2. This is in line with previous findings, but further subtypes the PTPR-B group. PTPR-A exhibited a distinct DNA methylation profile from PTPR-B1/B2, as well as a significantly higher genomic CNV load. While PTPR-A was shown to have significantly hypomethylated promoters relative to the B1/B2 groups, specific cancer-related biologic pathways were not identified in our analysis. Though the comparison of PTPR-B1 and B2 showed only subtle differences in terms of significantly different methylated levels of specific probes, the CNV profiles were highly distinct. As one example, PTPR-B2 was found to show predominantly normal copy numbers of chromosomes 3 and 14, while these two chromosomes were lost in PTPR-A and PTPR-B1 at frequencies of 60.0% and 91.7%, and 60.0% and 29.2%, respectively. While the epigenetic differences between PTPR-B1 and -B2 were subtle, the genomic differences, including the presence of chromosome 3 loss, which occurred in > 90% of -B1 cases but 0/31 -B2 cases. The clinical and biologic significance of this finding, however, is not clear and is an opportunity for further investigation. While we further found that PFS was different among the three subtypes, the sample size available for outcomes analysis was modest and more clinical outcome data is required to better understand these differences. As a preliminary finding the PFS time of PTPR-B2 was shorter (29 months) than the other two subtypes (> 60 months, *p* < 0.05), which might suggest that patients with PTPR-B2 tumors may warrant close clinical follow-up. Our refined PTPR tumor subtyping may thus serve to better tailor the clinical approach to patients with these tumors based on their genetic and epigenetic subtype and will hopefully stimulate more work in the study of this important tumor type.

### Supplementary Information

**Additional file 1**: UMAP plot of PTPR tumors. UMAP analysis used 10,000 highly variable DNA methylation probes by excluding probes from chromosomes 3, 9, 12, and 14.

**Additional file 2**: Supplementary data including Table S1: sample list and their clinical information, Table S2: promoter methylation results, and Table S3: chromosomal copy number variation results.

## Data Availability

Processed methylation results and raw data are available at the Gene Expression Omnibus (GEO) repository under the accession number GSE254031.

## Abbreviations

PTPR: Papillary tumor of the pineal region
GFAP: Glial fibrillary acidic protein
CNS: Central nervous system
CNV: Copy number variation
UMAP: Uniform Manifold Approximation and Projection
OS: Overall survival
PFS: Progression-free survival
