# Supplementary figures and images for: Papillary tumor of the pineal region: analysis of DNA methylation profiles and clinical outcomes in 76 cases
Source: Acta Neuropathol Commun. 2024 Jul 16;12:117. doi: 10.1186/s40478-024-01781-4 (PMC11251120; doi:10.1186/s40478-024-01781-4)

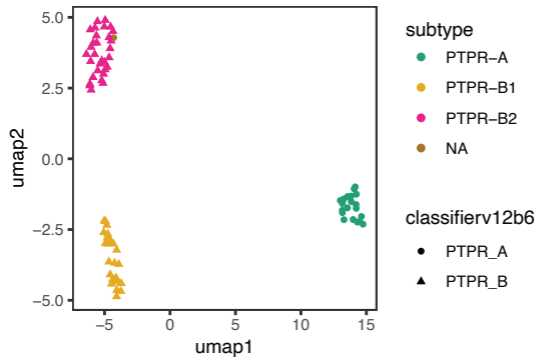

Supplement: Supplementary file 1 — Additional file 1: UMAP plot of PTPR tumors. UMAP analysis used 10,000 highly variable DNA methylation probes by excluding probes from chromosomes 3, 9, 12, and 14. [file 40478_2024_1781_MOESM1_ESM.pdf]
